# Microbial diversity as a foundation for biological AI: Learning biology from evolution’s largest dataset

**DOI:** 10.1038/s44319-026-00723-6

**Published:** 2026-03-09

**Authors:** Zachary N Flamholz, Libusha Kelly

**Affiliations:** 1 Department of Systems Biology, Albert Einstein College of Medicine, Bronx, NY 10641 USA; 2 Department of Microbiology and Immunology, Albert Einstein College of Medicine, Bronx, NY 10641 USA

**Keywords:** Computational Biology, Evolution & Ecology, Microbiology, Virology & Host Pathogen Interaction

## Abstract

The diversity of microbes reflects how cells adapt, interact and evolve across diverse environmental contexts and represents an unparalleled resource for training biological AI models. This microbial diversity can support models that go beyond sequence to capture and design biological function across systems.

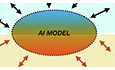

Microbes, meaning the prokaryotes, single-cell eukaryotes, archaea, and viruses are the Earth’s storehouse and historical record of the tremendous amount of genetic information that has evolved over millennia. Collectively, they represent a treasure trove of data not just on genes, proteins, and regulatory and metabolic pathways but also evolution, ecology, the interaction of diverse organisms, and their adaptation to environmental conditions. Yet, accessing and making sense of this wealth of data to answer fundamental biological questions is a major challenge despite ongoing large-scale sequencing efforts. It is here where AI holds great promise, given its ability to find meaningful patterns and make predictions from huge datasets. AI models are already being successfully employed for a wide variety of tasks, from predicting protein structure to gene annotation to metabolic pathway design.

We therefore propose microbes as simple and scalable model systems to represent a cell that has evolved under selective pressures to adapt to its environment and discuss how microbial genetic diversity can help to improve the performance and benefits of AI models. We argue that, with the help of AI, microbial sequence data from across our planet’s diverse environments can be used to address fundamental biological questions, such as evolution and adaption, how cells make decisions, the relationships and communication between organisms, and how emergent properties arise in biological systems. It would require multi-modal AI models that, in addition to sequence data, also incorporate additional ‘languages’ on environmental and evolutionary processes beyond linear DNA sequences.

“We therefore propose microbes as simple and scalable model systems to represent a cell that has evolved under selective pressures to adapt to its environment…”

## What is AI being used for in biology?

AI gained traction in biology by tackling a canonical problem: how a protein’s sequence encodes its structure and function. Solving this problem depends on representing biological sequences in a way that parallels how human language is represented in written text. The successful application of AI culminated in 2021 with the publication of AlphaFold at the 14th Critical Assessment of protein Structure Prediction (CASP14; Jumper et al, 2021) and set off a new era in protein modeling and generative design. One crucial aspect of AlphaFold was using multiple sequence alignments (MSAs) with atomic data and deep neural network models, such that structural and sequence-based features were refined together. Furthermore, AlphaFold showed that the number of sequences in a protein MSA is critical for predicting its structure.

In contrast, protein-language models (PLMs) can learn from diverse sequence data without relying on sequence alignments or known, or inferred, evolutionary relationships. Indeed, since AlphaFold, biological models have been trained on larger and larger databases of sequences: OMG (Cornman et al, 2024), EVO1 (Nguyen et al, 2024), or ESM3 (Hayes et al, 2025).

The ability of AI models to incorporate sequence data without the need for labels or annotations opens up novel ways to think about sequences in different contexts. As an example, multi-modal foundation models for human language, such as GPT-4, can be applied to a broad set of problems, including searching, writing documents, or creating images. Protein language models, such as ESM-2 and ProtT5, have analogous capabilities and have been used for tasks such as functional annotation (Flamholz et al, 2024), protein fold prediction (van Kempen et al, 2023) and predicting protein-DNA binding (Sanabria et al, 2024). Naturally, following proteins, DNA language models were explored. Evo2 is a foundation model for genome synthesis and design that was trained on ~9 trillion nucleotide sequences from ~ 113,000 bacterial and archaeal genomes and 15,000 eukaryotic genomes. The model can be used for a range of tasks, including predicting mutational effects on protein, RNA, and organismal fitness, creating genes and genomes, or designing sequences with specific chromatin accessibility properties (Brixi et al, 2025).

“The ability of AI models to incorporate sequence data without the need for labels or annotations opens up novel ways to think about sequences in different contexts.”

Following the achievements in structure and function prediction, it is logical to apply AI to other biological problems. One area that has attracted considerable effort and attention has been predicting cell perturbation effects, which are relevant to cell biology and human disease studies. Vast amounts of single-cell transcriptomic data have been collected to understand cellular diversity which, akin to the proliferation in genome and metagenome sequencing data, can serve to train AI models. Yet, in predicting the transcriptional effects of gene perturbations, single-cell foundation models perform no better than simpler statistical frameworks (Ahlmann-Eltze et al, 2025). While expression data likely contains patterns that could in fact enable prediction that surpasses linear regression, unlocking them may require new modeling strategies. Currently, single-cell transcriptomes have been primarily generated for eukaryotic cells, although approaches such as M3-seq (Wang et al, 2023) and microSPLiT (Gaisser et al, 2024) also enable it for bacterial cells. It is possible that the simpler transcriptional architecture of bacterial or archaeal cells may enable better prediction of the effects of gene perturbation in single-cell experiments.

## Designing better AI for biology

However, we are not there yet. Some of the failures of AI models to engineer functions and organisms or to address biological questions could be explained by the lack of critical information about evolution, environmental factors, and interaction among organisms and cells. For example, the failure of efforts to mitigate hyperammonemia suggests that even when you understand how to engineer a gut organism to perform a function, as in this case converting ammonia to l-arginine, it is not sufficient to drive the desired systemic phenotype.

There is substantial flexibility in the functions microbes perform, and the function that a cell chooses can be heavily influenced by the environment. And yet, for the sake of simplicity, we frequently label enzymes and microbes with single functions when an enzyme may have better or worse activity on various other compounds, and a microbe may produce butyrate but may also produce other metabolites that are critical for community or host health. If we tell an AI model that a certain organism is a ‘butyrate producer’, this reductionist annotation pigeonholes the model such that it only considers this bacterium as a butyrate producer.

Critically, all of these functions are dependent on the environment. While AI can incorporate vast unlabeled datasets in its models, labels are critical for prediction and will only become more important in AI training. This limitation means we may miss systemic properties that are not captured by the ‘primary’ function of a sequence. However, if we can combine function with the environment and communicate the context of functions to a model, we may get a better representation of reality; for example, a bacterium with the capacity to generate butyrate will generate butyrate if it has the right substrates, if it does not, it will not synthesize butyrate.

“… if we can combine function with environment and communicate the context of functions to a model, we may get a better representation of reality…”

Much of the excitement around the ability to predict protein structure and function lies in the generative application of such a tool. Combined with functional investigations into minimal genomes of bacteria (Hutchinson et al, 2016) and viruses (Adler et al, 2025), a generative approach would incorporate design into synthesis, ultimately helping to realize engineering of biologic entities. While this reality is no longer science fiction (King et al, 2025), and while AI will undoubtedly play a crucial role, the recycling of existing AI methods may only get us so far. If you want to generate a virus that will perform a certain action in the physical world, can stitching together DNA based on protein predictions achieve the biology you desire? Generative human monoclonal antibody design may serve as an informative lesson. Producing antibody sequences with complementary determining regions that bind epitopes on target antigens has been revolutionized by sequence-to-structure modeling. However, the ability of the antibody to function as a therapeutic requires other properties such as immunogenicity and toxicity that such models do not consider. As the bioscience community embraces AI, we must consider core assumptions of the technology that may or may not translate to solving a problem of interest.

## The power of microbial diversity for biological AI

Microbes are a storehouse of biological data over time and environments and are the source of the most diverse DNA sequences on the planet. They survive in wildly diverse environments, and, as a result of the diversity of ecosystem parameters, microbial cell structure, life-history strategies, and metabolism provide a broad map of life’s potential. Microbes are also tractable model systems for cellular structure and function that are amenable to genetic manipulation, high-throughput experimentation, and study in diverse environments. As such, they are a treasure trove of biological data for AI foundation models. Because AI models have the ability to see ‘all the data at once’, we propose that we can leverage microbial data to find unrecognized insights that govern fundamental biological processes across the tree of life.

“Because AI models have the ability to see ‘all the data at once’, we propose that we can leverage microbial data to find unrecognized insights that govern fundamental biological processes…”

Microbial DNA records evolution and environmental pressures and encodes constraints on community composition and ecology. For any protein that is shared across organisms, and there are many for which we cannot yet detect distant relationships, we have a record of the selective pressures on that protein sequence at a cellular and environmental level over time that is encoded in its sequence. Can AI use this data to find patterns, for example, in proteins that represent temperature, or pH or nutrient limitations? If so, one could use that information to guide studies that seek to design proteins that work under specific environmental conditions: in a high-heat recycling plant, or in wastewater treatment, or in the anoxic environment of a tumor.

Beyond annotation, one could imagine that enzyme design could be improved by better representations of the evolutionary landscape of a functional protein of interest. Explicitly incorporating microbial diversity, either in training, or in tuning models for problems such as “design a plastic-degrading enzyme that works under the high-heat conditions of a recycling plant” could implicitly take advantage of protein sequence data from thermophilic organisms, not just restricted to structurally similar enzymes, to learn sequence features that augment the ability of an enzyme to perform at high temperatures.

## New languages for biology

The most abundant raw data that we currently have in biology is DNA sequence data. One assumption of current models is that alphabets are appropriate symbolic representations, including both the DNA and protein sequence alphabets—the As, Cs, Ts, Gs, Ks, Ts and so on – and symbolic alphabets, such as the Prot5 representation of the side chain properties of amino acids (Elnaggar et al, 2022) or the structure-to-sequence 3Di alphabet of ProstT5 (Heinzinger et al, 2024).

However, we know there are other features that play a key role in structuring microbial communities and their functions. Environment is critical, and aggregated, detailed data on environmental parameters are available for metagenomic samples in resources such as Metalog (Kuhn et al, 2026). The evolutionary history of sequences is another place where information on function is stored. Finally, interactions structure microbial communities, including, but not being limited to, metabolic, physical, and chemical interactions. Representation of this dynamic network of information exchange in microbial communities is lacking in current AI models. To include this critical data, we must step past sequence representations of microbial life and design new models with these datatypes in mind. Inevitably, this would require multi-modal AI models that use a range of diverse datatypes similar to foundational LLMs.

“To include this critical data, we must step past sequence representations of microbial life and design new models with these datatypes in mind.”

Indeed, the interactions among biological entities are a major missing piece of contemporary AI models. Instead of alphabets, one could represent environmental, evolutionary, and timeseries data of interactions between biological elements as networks. Such interaction networks might include physical or killing interactions, such as a phage infecting a host; the transfer of nucleotide resources via horizontal exchange, mobile elements, phages, and vesicles; or the exchange of metabolites. These interactions are fundamental to life and could be described across systems and scales. As an example, despite differences between bacterial and human cells, we can perhaps learn how biological entities make decisions and function at scale using bacteria. Bacterial cell biology can be scaled to AI; high-throughput genetics methods exist to identify triggers or blockers of bacterial toxin-antitoxin systems to identify interactions (Bobonis et al, 2024).

“Instead of alphabets, one could represent environmental, evolutionary and timeseries data of interactions between biological elements as networks.”

Training data for such models may not necessarily require the giant corpuses used to train LLMs. In training an encoder neural network model to predict antimicrobial peptides only ~25,000 examples were used; the resulting model outperformed simple machine-learning predictors (Wan et al, 2024). The majority of the experimental data used for training ( ~ 14,000 examples) was generated under the control of a single lab, speaking to the potential for high-quality data to enable complex models. High-quality data from other medium-throughput datasets such as the phage/host interaction network described in Kauffman et al, (2022) could similarly be used to train models to predict phage/host interactions, for example, by encoding all phage and host genes and predicting a killing or phage entry interaction, with the performance of AI models compared to simpler models.

A widely used training approach in current AI models includes masked token objectives in which input tokens, meaning the until of modeling such as an amino acid in a protein language model or transcript count in a cell foundation model, are ‘masked’, or blocked out, and the model’s objective is to predict the masked token. Reinforcement learning with human feedback is another common approach in which a model is trained with feedback from human users, for example, in the case of designing a protein to bind a specific small molecule.

**Figure 1:**
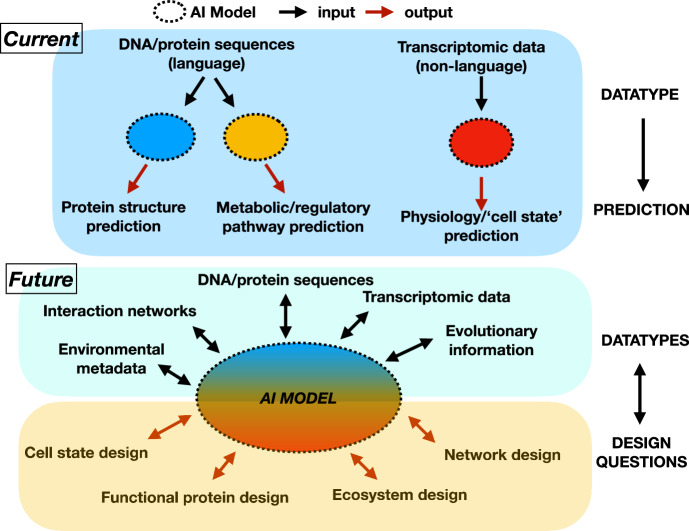
Future AI models for biology and biomedicine. Current models (top) generally take a single datatype as input, for example, sequences or transcriptomic data, and produce predictions or downstream design tasks. Future models (bottom) could produce designs based on multiple datatypes to address broader questions with feedback between data and design. As an example, imagine we want to design plastic-degrading enzymes to work in the high-temperature environment of a plastic recycling plant. In the current single-datatype model, we would take sequences with known plastic-degrading functions and use an AI model to predict additional sequences that can carry out this desired function, with a focus on searching high-temperature environments, and subsequently use the same model again to design newly identified enzymes for better activity on the desired plastics. Notice that here we do not consider the microbial chassis that will carry the enzyme, only the enzyme itself. In a future multi-modal model, we could input information about environmental constraints, taxonomic constraints, and sequences, and get back designed microbes and microbial communities that perform high-throughput breakdown of plastics at high temperature. We not only design the enzyme, but the bacterium in a community that will support its production of the enzyme in the recycling plant environment.

Future objectives in the same vein might include prediction of a missing function from a community or a missing interaction that enables production of a metabolite in a community; however because the objective is fundamental to how models are trained, new objectives for biological questions may be required. For example, can we identify the next, most informative experiments for lab-in-the-loop approaches? Can we use high-throughput screening to identify 10 novel enzymes that degrade a plastic and can we predict which are the most promising candidates to test in a real-world scenario for this desired function? Such approaches highlight a powerful aspect of AI: it will reflect back to you how well or poorly you’ve defined your problem (Fig. 1).

## Data collection for biological AI

High-throughput experimentation can collect data specifically to improve AI models. One could envision a sequencing study to this end: one could first perform shallow shotgun sequencing to compare the new data to all environments ever sequenced. One could then go deeper if the sample is significantly different from what has already been seen. TARA, GOS or other large environmental metagenomic datasets could be used for preliminary power calculations for such an effort by rarefying protein sequences and asking how many samples in a test set have high protein sequence overlap with other samples and therefore do not need further sampling, versus samples with low sequence overlap that could be sampled more deeply or to flag a particular environment for more sequencing due to novelty.

Michael Bronstein, founding scientific director of the Aithyra Institute, has proposed data collection by “black box” modalities that are not directly interpretable by human users but are rather developed in concert with AI models that use the data to improve predictions in a domain of interest (Lemberger, 2024). Indeed, microbes are well-suited for such efforts given their small and simpler genomes, abundance, and the enormous diversity and complexity they represent along with the availability of richly phenotyped and annotated genomes. A danger here is that microbes that are not high-throughput friendly will be underrepresented in such studies. Interactions between and cooperation among the computational side of such lab-in-the-loop models and biologists who are versed in microbial ecosystems will be critical for progress.

Once we have these new models what can we do with them? We could design microbes that act as sensors for specific molecules in the human body, for example designing a gut-colonizing bacteria to read out the presence of high sulfide concentrations. In ecosystems at risk from climate change, a microbe could exude chemical signals related to temperature or pH changes. We could design immune system-modulating microbes to improve human health, or design phages as hyper-targeted antibacterials.

## Trusting the answers

A recent paper (Ahdritz et al, 2024) demonstrated that an open, trainable implementation of AlphaFold2 learns structural components hierarchically. This after-the-fact insight demonstrates one major limitation of current PLMs: these are ‘black box’ models, with little insight into what the model is ‘doing’. This obscured view is in contrast with BLAST, a tool that one of us was given the task of coding in their very first bioinformatics class, and that the majority of our community uses. BLAST remains the first place many scientists go when they ask the question: I have this protein, what does it do? The comforting thing about BLAST is that you can look under the hood of the model because its score is based on alignments; looking at your protein’s alignment with other proteins provides an intuition of what the algorithm is doing. In contrast, PLMs are less intuitive and currently much more of a black box tool where the score is not easily traced back to a biological feature.

With a structured training approach, where we, for example, leave out sets of data, we can address the question of what the model is representing to ask if there is an order, or hierarchy, to how it identifies features of interest. An alternative approach could include training protocols that incorporate user input to steer the model towards desired outcomes; however, this carries the risk of stifling the ability of the model to generalize and instead encourages it to identify things we already know. Such studies could vastly improve how we train, or tune, models for biological questions and, more broadly, can start to crack open the logic of representations to enable scientists to use them in novel ways. Moreover, model validation is critical for trust. Akin to standard machine-learning protocols that train, test, and validate models, one must utilize appropriate, unseen data from a different source and system than the training/testing set to assess performance.

The AI models described here are, by their very nature and design, not conceptual. In fact, the next generation of models, such as BioReason and Kosmos, are trained to ‘reason’ about scientific data, that is, to take language-based questions like “what disease might be caused if gene X is mutated?” and deliver interpretable reasoning about their responses (https://arxiv.org/abs/2505.23579; https://arxiv.org/abs/2511.02824).

## We are all systems biologists now

A wealth of publicly accessible and simple AI tools has allowed researchers to benefit from and discover novel biology: ColabFold, Foldseek, and other tools have greatly advanced research. Yet, as anyone who uses them can tell you, AI approaches are not magic. Like all models, they are biased by their training data, noisy, and spit out plausible but factually incorrect statements. Such models lend themselves to hypothesis generation and, due to their generality, may take effort away from validation. Annotated sets of tens of thousands of annotated human gut microbiomes have not been enough to give us a general, quantitative, and actionable definition of human gut health; another 100,000 metagenomes won’t fix that problem. So, somewhat counterintuitively, biological AI is not a task that can be fulfilled with just more data alone.

At their best, AI models will help biologists bring to life Carle Woese’s call to action more than 20 years ago: “our task now is to resynthesize biology; put the organism back into its environment; connect it again to its evolutionary past; and let us feel that complex flow that is organism, evolution, and environment united. The time has come for biology to enter the nonlinear world.” And yet, we also run the risk of what Woese saw as a defining failing of microbiology in the 1900s, “a prime example of a science not seeking to define itself, letting itself instead be defined by external influences”, being turned into nothing more than a “technological playground” in service to practical needs (Woese, 2004). Turning AI over to the domain of ‘data science’ and computer science does a disservice to scientists and raises the disturbing possibility that science will be harnessed in the service of AI rather than AI being harnessed in the service of science.

“Turning AI over to the domain of ‘data science’ and computer science does a disservice to scientists and raises the disturbing possibility that science will be harnessed in service of AI rather than AI being harnessed in service of science.”

That is why we need to train students of biology to effectively use AI to advance biology instead the other way around. Their training should combine rigorous hypothesis-generation and testing with exploratory data analyses to leverage and master the ability of AI to uncover hidden connections. AI’s great strength is in looking at all the data at once and finding unrecognized patterns in a specific dataset (Flamholz et al, 2024), and this ability is helpful for addressing broader problems across biological fields. To do so, however, we must encourage our trainees, and ourselves, to step out of our individual scientific silos and go to talks in other fields and to act on the little spark in the brain that comes when we see things from a different perspective. And so, in a commentary on AI models in biology, we end by encouraging students and scientists alike to engage with human connections and the natural world to make new discoveries in biology and biomedicine—with a little help from our AI models.

## Supplementary information

Peer Review File
