# Supplementary material for: Microbial diversity as a foundation for biological AI: Learning biology from evolution’s largest dataset
Source: EMBO Rep. 2026 Mar 9;27(7):1642–7. doi: 10.1038/s44319-026-00723-6 (PMC13076786; doi:10.1038/s44319-026-00723-6)
Supplement: Supplementary file 1 — Peer Review File [file 44319_2026_723_MOESM1_ESM.pdf]

EMBO reports

Microbial diversity as a foundation for biological AI

Zachary Flamholz and Libusha Kelly

Corresponding author(s): Libusha Kelly (libusha.kelly@einsteinmed.edu)

**Review Timeline:**	Submission Date:	8th Feb 26
	Accepted:	9th Feb 26

Editor: Holger Breithaupt

Transaction Report: This article was editorially reviewed.
